# Synthesis and Anticancer Activity of New 1-Thia-4-azaspiro[4.5]decane, Their Derived Thiazolopyrimidine and 1,3,4-Thiadiazole Thioglycosides

**DOI:** 10.3390/molecules22010170

**Published:** 2017-01-20

**Authors:** Eman M. Flefel, Wael A. El-Sayed, Ashraf M. Mohamed, Walaa I. El-Sofany, Hanem M. Awad

**Affiliations:** 1 Department of Chemistry, College of Science, Taibah University, Al-Madinah Al-Monawarah 1343, Saudi Arabia; emanmflefel@yahoo.com; 2 Photochemistry Department, National Research Centre, Dokki 12622, Giza, Egypt; 3 Applied Organic Chemistry Department, National Research Centre, Dokki 12622, Giza, Egypt; ammewas1@gmail.com; 4 Chemistry Department, College of Science, Aljouf University, Sakaka, Al-Jouf 72341, Saudi Arabia; 5 Department of Tanning Materials and Leather Technology, National Research Centre, Dokki 12622, Giza, Egypt; hanem_awad@yahoo.com

**Keywords:** 1-thia-azaspiro[4.5]decane, thiazolopyrimidine, thioglycosides, 1,3,4-thiadiazoles anticancer activity

## Abstract

New 1-thia-azaspiro[4.5]decane derivatives, their derived thiazolopyrimidine and 1,3,4-thiadiazole compounds were synthesized. The thioglycoside derivatives of the synthesized (1,3,4-thiadiazolyl)thiaazaspiro[4.5]decane and thiazolopyrimidinethione compounds were synthesized by glycosylation reactions using acetylated glycosyl bromides. The anticancer activity of synthesized compounds was studied against the cell culture of HepG-2 (human liver hepatocellular carcinoma), PC-3 (human prostate adenocarcinoma) and HCT116 (human colorectal carcinoma) cell lines and a number of compounds showed moderate to high inhibition activities.

## 1. Introduction

Chemotherapy is considered as an important option [1] among applied strategies for cancer treatment and the major objective of many approved chemotherapeutic agents [2], is the induction of apoptosis of cancer cells. Research for scouting novel selective anticancer agents, with minimal side effects, is a demanding requirement because of toxicity to normal cells, decreased drug activity and development of drug resistance, which are responsible for insufficiency in cancer treatment.

It has been found that the thiazolidine ring system is mainly integrated in important compounds with interesting applications in medicinal and pharmaceutical chemistry. A variety of established drugs and drug candidates such as Pioglitazone, Epalrestat, Letosteine, and Tidiacichave, have in their skeleton, a thiazolidine nucleus. Such a nucleus is described as a wonder moiety because of the wide spectrum of biological activities associated with its derivatives. The N–C–S linkage had been found to be responsible, in active compounds, for exhibiting antimicrobial [3,4,5] and anti-HIV [6,7] activities. The thiazolidine nucleus, notably 1,4-thiazolidinedione (TZD), has been widely employed as a unique scaffold in developing new potent anticancer agents showing cytotoxicity against different human cancer cells [8,9,10] and recently was intensified as a novel onset in cancer chemotherapy [11,12]. Efatutazone, netoglitazone, rosiglitazone and troglitazone, having the thiazolidine system in their basic skeleton, are being studied regarding the mechanism underlying their anticancer activity [11].

**Figure 1:**
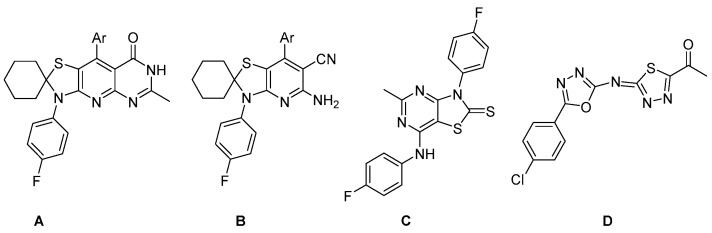
Anticancer spiro-thiazolidine (**A** and **B**), thiazolopyrimidine (**C**) and 1,3,4-thiadiazole (**D**) compounds.

Although spiro-compounds allow conformational qualifications and original orientation of the diversity of elements, spirothiazolidines have been poorly investigated. This scaffold is believed to be a precious isostere for known active spiroimidazolidines, spirohydantoines or spirobenzofuranes, with implementations in G protein–coupled receptors (GPCRs) and peptidomimetics. It is incorporated in *Spiclomazine*, an antipsychotic drug. Simple spirothiazolidines have been revealed for their inhibition activity of metalloprotease which allows their application in the control of malaria infection [13]. Spirothiazolidine derivatives were also found to be of good activity as anticancer and antioxidant [14] agents. Thiazolopyrimidine is an interesting bicyclic system as its derivatives revealed their activity as analgesic and antiparkinsonian agents [15], Transient Receptor Potential Vanilloid–receptor 1 (TRPV1) modulators [16], anticancer agents [17,18,19,20], antioxidants [21], pesticides [22], phosphate inhibitors [23,24], acetylcholinesterase inhibitors [25] and antimicrobial substances [26,27,28]. Structures **A**–**D** (Figure 1) with spiro-thiazolidine, thiazolopyrimidine and 1,3,4-thiadiazole ring systems have been reported with their anticancer activity [14,29,30].

Thioglycosides and their analogs incorporating modified glycosyl and/or aglycon parts have prompted extended research as biological inhibitors [31,32,33]. A number of thioglycosides have been reported with their potential biological activities in addition to their use as glycosyl donors [32,33,34,35]. The above findings and our continued research program targeting synthesis of new glycosyl heterocycles [33,35,36,37,38] and their acyclic analogs with anticancer activity have prompted us to synthesize new thia-azaspiro[4.5]decane, their derived thiazolopyrimidine and 1,3,4-thiadiazole thioglycosides with anticancer evaluation studies.

## 2. Results and Discussion

### 2.1. Chemistry

The thia-4-azaspiro[4.5]decan compounds **1**–**3** were prepared via a one-pot three-component reaction involving condensation of ketones namely, 4-methylcyclohexanone and cyclohexanone; aromatic amines namely, 4-bromoaniline or 4-fluoroaniline; and mercaptoacetic acid in dry benzene to afford the corresponding substituted aryl-1-thia-4-azaspiro[4.5]decan-3-one compounds **1**–**3** [14]. The Infra-Red spectra of the resulting spiro-thiazolidine derivatives **1** and **3** showed characteristic peaks at 1682 and 1677 cm^−1^ corresponding to the thiazolidinone carbonyl function. The ^1^H-NMR spectra of compounds **1** and **3** displayed a singlet signal at 0.88 and 0.86 ppm to methyl protons while the signal attributed to methylene protons of the thiazolidinone ring appeared at 3.39 and 3.37 ppm. Their ^13^C-NMR spectrum showed characteristic peaks corresponding to the C=O group and the signals assigned to the spiro-C atom. Condensation of the fluoro-substituted derivative **2** with 2-methylfurfural in ethanol under reflux in the presence of a base led to the arylidene derivative **4** formation in 74% yield. The IR spectrum of the afforded furyl-arylidene **4** showed the band at 1672 cm^−1^ corresponding to the C=O group. Its ^1^H-NMR spectrum revealed the disappearance of the signals of the thiazolidine-CH_2_ protons and existence of signals assigned to the methine and aryl hydrogens. The ^13^C-NMR spectrum showed signals at δ 73.35 for the spiro-carbon, 116.4–160.1 corresponding to the sp^2^ carbons and 167.9 for the carbonyl group. When compound **4** was fused with thiourea in the presence of a catalytic amount of piperidine at 180 °C, it afforded the thiazolo[4,5-*d*]pyrimidine]-5′(6′*H*)-thione derivative **5** for which the obtained spectral characteristics confirmed its assigned structure. The ^1^H-NMR spectrum showed the signal at 13.25 for NH tautomerized with C=S in addition to cyclohexyl and aryl protons.

**Scheme 1:**
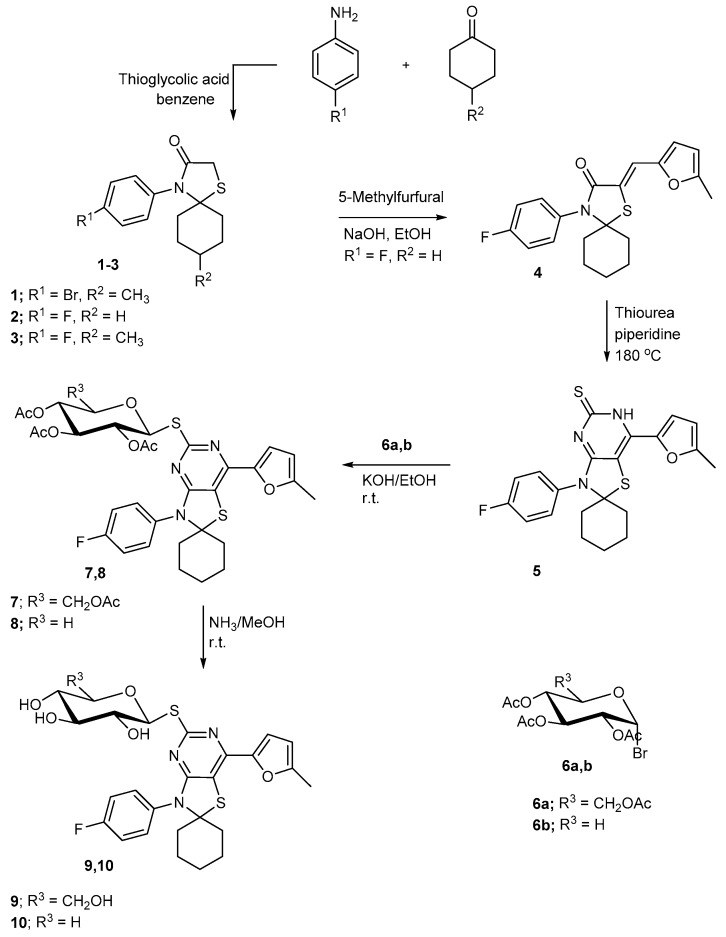
Synthesis of spiro[cyclohexane-1,2′-thiazolo[4,5-*d*]pyrimidine] glycosides.

The reaction of the thiazolopyrimidinethione derivative **5** with 2,3,4,6-tetra-*O*-acetyl-α-d-gluco- or 2,3,4,-tri-*O*-acetyl-α-d-xylopyranosyl bromide **6a**,**b** afforded the corresponding glycosylthio-derivatives of the thiazolopyrimidine nucleus **7** and **9**, respectively. The ^1^H-NMR spectra of the resulting thioglycosides showed the signal corresponding to acetyl methyl protons in addition to the other protons of the sugar chain. The formation of these thioglycosides and linkage of the sugar part at a sulfanyl center was confirmed by the NMR data. The relatively low chemical shift of the anomeric proton (H-1) in the ^1^H-NMR and the absence of the C=S signal in the ^13^C-NMR spectra indicates such a mode of attachment. In *N*-glycosides, the anomeric proton is known to be found at relatively higher chemical shift values (5.95–6.15) [39,40] as a result of the deshielding influence of the thione function. The coupling constant of the anomeric proton also revealed the β-thioglycosidic linkage of the sugar moiety in the produced thioglycosides. Deacetylation of the latter glycosides **7** and **8** with methanolic ammonia solution led to the formation of the corresponding free hydroxyl derivatives **9** and **10**, respectively (Scheme 1). Their IR spectra indicated the disappearance of acetyl carbonyl groups and the appearance of characteristic peaks in the hydroxyl frequency area attributed to sugar hydroxyls and the ^1^H-NMR spectra are agreeing with their assigned structure.

**Scheme 2:**
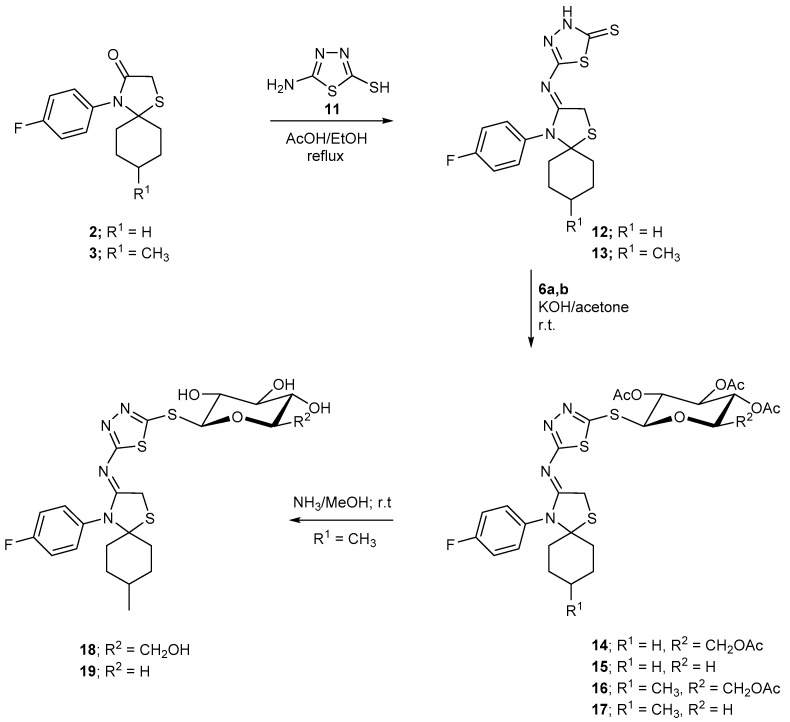
Synthesis of 1,3,4-thiadiazole thioglycoside.

Nucleophilic addition between primary amine and carbonyl aldehyde/ketone to prepare Schiff bases is also common for amino-substituted thiadiazoles [41,42]. The pair of electrons of the nitrogen in the NH_2_ allows the nucleophilic substitution process to be easily performed [43]. Condensation of compounds **2** and **3** with 2-amino-1,3,4-thiadiazole-5-thiol (**11**) in ethanol gave the corresponding arylidine-1,3,4-thiadiazole-2-thione derivatives **12** and **13**, respectively. The absence of the carbonyl band in the IR spectra and the presence of the NH group in their ^1^H-NMR spectra confirmed the assigned structures. Glycosylation of the afforded latter 1,3,4-thiadiazole derivatives via reaction with *O*-acetyl-α-d-gluco- or pyranosyl bromide compounds **6a**,**b**, led to the formation of the corresponding 1,3,4-thiadiazole thioglycoside derivatives **14**–**17** in 65%–75% yields. The IR spectral data showed the bands corresponding to the glycosyl acetyl carbonyl groups in the C=O frequency zone. The chemical shift values and the coupling constant values in their obtained NMR spectral data have revealed the β-glycosidic linkage nature at the sulfur atom in the form of 1,3,4-thiadiazole thioglycosides. The deportation (deacetylation) reaction was carried out for two of the latter synthesized thioglycosides; one has the glucopyranosyl moiety (six carbon sugar part) and the other incorporates a xylopyranosyl unit (five carbon glycosyl). Thus, the free hydroxyl thioglycosides of the (thiadiazolyl)thia-4-azaspiro[4.5]decan incorporating the substituted cyclohexyl ring **18** and **19** were obtained by deacetylation reactions of the their acetylated precursors with methanolic ammonia solution (Scheme 2). The absence of the acetyl carbonyl bands in the IR spectra of the latter unprotected thioglycosides, in addition to the appearance of the hydroxyl absorption bands confirmed the assigned structures. Furthermore, their ^1^H-NMR spectra showed signals corresponding to the sugar hydroxyls and chain protons, in addition to the disappearance of the acetyl-methyl signals.

### 2.2. Anti-Tumor Activity

**Figure 2:**
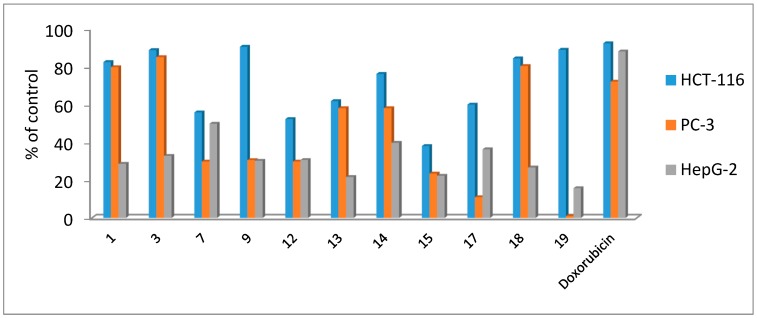
Anticancer activity of compounds against human liver hepatocellular carcinoma (HepG-2), human prostate adenocarcinoma (PC-3) and human colorectal carcinoma (HCT-116) cell lines using a 3-[4,5-dimethyl-2-thiazolyl)-2,5-diphenyl-2*H*-tetrazolium bromide (MTT) assay at 100 ppm.

The synthesized compounds were examined in vitro for their anti-tumor activities against human liver hepatocellular carcinoma (HepG-2), human prostate adenocarcinoma (PC-3) and human colorectal carcinoma (HCT-116) cell lines using a MTT assay. The percentage of the intact cells was measured and compared to the control (Figure 2). The IC_50_ values of tested compounds are shown in Table 1.

The activities of these compounds against the three carcinoma cells were compared with that of Doxorubicin^®^.

The obtained results showed that all compounds showed dose-dependent anticancer activities against the three cancer cells. Compounds which did not show anticancer activity against the previously mentioned cell lines at concentration 100 ppm or more have not been investigated for IC_50_ values. The results outlined in Figure 2 and Table 1 indicated that five compounds (**7**, **9**, **14**, **18** and **19**) showed good anticancer activities against HCT-116 carcinoma cells with IC_50_ ranging from 92.2 to 120.1 nM. The rest of the compounds showed moderate activities against HCT-116 cells.

The activity results against PC-3 cancer cells revealed that compounds **14** and **18** showed good anticancer activities. In addition, five compounds (**1**, **3**, **7**, **9** and **13**) showed moderate inhibition activities against this type of cancer cells and the rest of the compounds showed weak activities. Furthermore, four compounds (**7**, **14**, **17** and **9**) showed moderate activities and the rest of the compounds showed weak anticancer activities against HepG-2 liver cancer. 

Depending on the above results and on their correlation with the structures of highly active compounds, the obtained inhibition activity results of compounds **7**, **9**, **14**, **17**–**19** revealed the importance of attachment of glycosyl moieties to the thiazolopyrimidine or (1,3,4-thiadiazolyl)thiazolidinone ring systems. It is clear that the activity was raised in the latter compounds in which the polyhydroxy or poly-acetylated cyclic sugar unit was linked via a thioglycosidic linkage to the 1,3,4-thiadiazole or thiazolopyrimidine ring systems compared to the glycosyl free heterocycles **5**, **12** and **13**. This conclusion was also supported by the obtained results for compound **4**, with little activity against the previously mentioned cells, in which the thiazolidine ring was substituted with methylfurane substituent. Furthermore, the attachment of glucopyranosyl moiety to the thiazolopyrimidine or 1,3,4-thiadiazol ring system (compounds **7**, **9**, **14** and **18**) led to an increase in inhibition activity more than the presence of xylopyranosyl moiety. 

On the other hand, it can also be concluded that the attachment of the alkyl group at position-4 in the cyclohexyl ring in the tested spiro-compounds resulted in high activity against HCT-116 and PC-3 cancer cells. This is clear as the activity was decreased in compounds **2** and **12** in which the cyclohexyl group is free of substitution with methyl substituent. 

## 3. Materials and Methods

### 3.1. Chemistry

All melting points are uncorrected and were determined on a Stuart electric melting point apparatus. The IR spectra were recorded on a Thermo Nicolet (Thermo Scientific, Madison, WI, USA) using KBr disks. The NMR spectra (500/125 MHz) were determined by using a Bruker NMR spectrometer (Rheinstetten, Munchen, Germany). A chemical shift was expressed in δ (ppm) downfield from tetramethylsilane (TMS) as an internal standard. Mass spectra were recorded at 70 eV EI Ms-QP 1000 EX (Shimadzu, Kyoto, Japan). The microanalysis was within ±0.4% of the theoretical values and was carried out at the Microanalytical Centre, National Research Centre, Cairo, Egypt. Homogeneity of all compounds synthesized was checked by thin layer chromatography (TLC) which was performed on Merck 60 (Munchen, Germany) ready-to-use silica gel plates to monitor the reactions and test the purity of the new synthesized compounds. The chemical names given for the prepared compounds are according to the International Union of Pure and Applied Chemistry (IUPAC) system.

#### 3.1.1. 8-Substituted-4-aryl-1-thia-4-azaspiro[4.5]decan-3-one (**1**–**3**)

A mixture of cyclohexanone or methylcyclohexanone (0.01 mol), aromatic amine, namely, 4-bromoaniline or 4-flouroaniline (0.01 mol), and thioglycolic acid (0.01 mol, 0.92 mL) in dry benzene (50 mL) was refluxed for 10 h. The solution was concentrated; the formed solid was filtered off, dried, and crystallized to give compounds **1**–**3**.

*4-(4-Bromophenyl)-8-methyl-1-thia-4-azaspiro[4.5]decan-3-one* (**1**). White powder, Yield: 77%; m.p. 130–131 °C; IR (KBr, υ, cm^−1^): 3055 (C-H aromatic), 2925 (C-H aliphatic), 1682 (C=O); ^1^H-NMR (DMSO-*d*_6_): δ (ppm) 0.88 (d, *J* = 6.2 Hz, 3H, CH_3_), 1.24–1.77 (m, 9H, cyclohexyl-H), 3.39 (s, 2H, CH_2_), 7.20 (d, *J* = 7.6 Hz, 2H, Ar-H), 7.32 (d, *J* = 7.6 Hz, 2H, Ar-H); ^13^C-NMR: 23.5–39.4 (5CH_2_), 43.8 (CH_2_), 74.2 (spiro-C), 115.4–154.1 (Ar-C), 172.2 (C=O); MS, *m*/*z* (%): 339 (M^+^ + 28), 341 (M^+^ + 2, 26). Analysis calc. for C_15_H_18_BrNOS (340.28): C, 52.95; H, 5.33; N, 4.12. Found: C, 52.74; H, 5.19; N, 4.19.

*4-(4-Fluorophenyl)-1-thia-4-azaspiro[4.5]decan-3-one* (**2**) [14]. This compound was prepared as reported earlier as pale yellow needle crystals; m.p. 142–143 °C (Reported m.p. 143–145 °C).

*4-(4-Fluorophenyl)-8-methyl-1-thia-4-azaspiro[4.5]decan-3-one* (**3**). Pale yellow powder, Yield: 72%; m.p. 125–126 °C; IR (KBr, υ, cm^−1^): 3058 (C-H aromatic), 2930 (C-H aliphatic), 1677 (C=O); ^1^H-NMR (DMSO-*d*_6_): δ (ppm) 0.86 (d, *J* = 6.4 Hz, 3H, CH_3_), 1.24–1.79 (m, 9H,5CH_2_), 3.37 (s, 2H, CH_2_), 7.22 (d, *J* = 7.8 Hz, 2H, Ar-H), 7.34 (d, *J* = 7.8 Hz, 2H, Ar-H); ^13^C-NMR: 23.1 (CH_3_), 24.0–39.5 (cyclohexyl-CH_2_ and -CH), 43.7 (CH_2_), 74.1 (spiro-C), 115.4–161.1 (Ar-C), 172.3 (C=O); MS, *m*/*z* (%): 279.3 (M^+^, 35). Analysis calc. for C_15_H_18_FNOS (279.37): C, 64.49; H, 6.49; N, 5.01. Found: C, 64.21; H, 6.31; N, 5.22.

#### 3.1.2. 4-(4-Fluorophenyl)-2-((5-methylfuran-2-yl)methylene)-1-thia-4-azaspiro[4.5]decan-3-one (**4**)

A mixture of compound **2** (0.01 mol, 2.65 g) and 5-methylfurfural (0.01 mol, 1.1 g) in ethanolic sodium hydroxide solution (10%, 25 mL) was heated under reflux for 6 h. The reaction mixture was poured onto ice-cold water (50 mL) and neutralized with dilute HCl; then the formed solid was filtered off and crystallized from ethanol to give the arylidine derivative **4**.

Brownish-red powder, Yield: 74%; m.p. 122–123 °C; IR (KBr, υ, cm^−1^): 3045 (C-H aromatic), 2940 (C-H aliphatic), 1672 (C=O); ^1^H-NMR (DMSO-*d*_6_): δ (ppm) 1.42–1.96 (m, 10H, 5CH_2_), 2.41 (s, 3H, CH_3_), 6.30 (d, *J* = 7.8 Hz, 1H, furyl-H), 6.60 (d, *J* = 7.8 Hz, 1H, furyl-H), 7.38 (s, 1H, C*H*=C), 7.20 (d, *J* = 8.2 Hz, 2H, Ar-H), 7.36 (d, *J* = 8.2 Hz, 2H, Ar-H); ^13^C-NMR: 21.4 (CH_3_), 24.1–39.37 (5CH_2_), 73.35 (spiro-C), 116.4–160.1 (Ar-C and methine-C and thiazolidine-C5, 167.9 (C=O); MS, *m*/*z* (%): 357.12 (M^+^, 26.5). Anal. calc. for C_20_H_20_FNO_2_S (357.44): C, 67.21; H, 5.64; N, 3.92. Found: C, 67.02; H, 5.51; N, 3.69.

#### 3.1.3. 3′-(4-Fluorophenyl)-7′-(5-methylfuran-2-yl)-3′*H*-spiro[cyclohexane-1,2′-thiazolo[4,5-*d*]pyrimidine]-5′(6′*H*)-thione (**5**)

A mixture of the compound of the arylidine **4** (0.01 mol, 3.57 g) and thiourea (0.01 mol, 0.76 g) was fused in the presence of 10 drops of piperidine in an oil bath for 3 h at 180 °C. The product was poured onto crushed ice; the solid was filtered off and recrystallized from acetic acid to give compound **5**.

Brownish powder; Yield: 74%; m.p. 140–141 °C; IR (KBr, υ, cm^−1^): 3241 (NH), 3045 (C-H aromatic), 2940 (C-H aliphatic), 1612 (C=N); ^1^H-NMR (DMSO-*d*_6_): δ (ppm) 1.64–1.96 (m, 10H, 5CH_2_), 2.46 (s, 3H, CH_3_), 6.70 (d, *J* = 7.8 Hz, 1H, furyl-H), 6.90 (d, *J* = 7.8 Hz, 1H, furyl-H), 7.26 (d, *J* = 8.2 Hz, 2H, Ar-H), 7.60 (d, *J* = 8.2 Hz, 2H, Ar-H), 13.25 (brs, 1H, NH); ^13^C-NMR: 21.4 (CH_3_), 23.8–39.4 (5CH_2_), 73.35 (spiro-*C*), 116.38–161.4 (Ar-C and pyrimidine-C5,6), 165.2 (C=N), 182.13 (C=S); MS, *m*/*z* (%): 413.2 (M^+^, 1.37). Anal. calc. for C_21_H_20_FN_3_OS_2_ (413.53): C, 60.99; H, 4.88; N, 10.16. Found: C, 60.72; H, 4.75; N, 9.90.

#### 3.1.4. 3′-(4-Fluorophenyl)-7′-(5-methylfuran-2-yl)-5′-(*O*-acetyl-d-glycopyranosyl)-3′*H*-spiro[cyclohexane-1,2′-thiazolo[4,5-*d*]pyrimidine] (**7**,**8**)

To a well stirred solution of thione derivative **5** (0.005 mol, 2.65 g) in aqueous potassium hydroxide [0.005 mol in distilled water (2 mL)] was added a solution of 2,3,4,6-tetra-*O*-acetyl-α-d-gluco- or 2,3,4,-tri-*O*-acetyl-α-d-xylopyranosyl bromide (**6a**,**b**) (0.005 mol) in acetone (20 mL)portion wise. The reaction mixture was stirred at room temperature for 6 h at which TLC (pet. ether/EtOAc 3:1) showed completion of the glycosylation. The solvent was evaporated under reduced pressure and the residue was washed with distilled water to afford a product which was dried and crystallized from ethanol to give compound **7** and **8**, respectively.

*3′-(4-Fluorophenyl)-7′-(5-methylfuran-2-yl)-5′-(2,3,4,6-tetra-O-acetyl-d-glucopyranosyl)-3′H-spiro[cyclohexane-1,2′-thiazolo[4,5-d]pyrimidine]* (**7**). Brownish powder, Yield: 76%; m.p. 129–130 °C; IR (KBr, υ, cm^−1^): 3055 (C-H aromatic), 2950 (C-H aliphatic), 1748 (C=O), 1614 (C=N); ^1^H-NMR (DMSO-*d*_6_): δ (ppm) 1.51–1.91 (m,10H, 5CH_2_), 1.97, 2.06, 2.11, 2.18, 2.35 (5s, 15H, 5CH_3_), 3.70–3.75 (m, 1H, H-5′), 4.02–4.07 (dd, *J* = 3.8, 10.2 Hz, 1H, H-6′′), 4.15–4.19 (dd, *J* = 11.3, 3.8 Hz, 1H, H-6′), 4.60 (t, *J* = 8.6 Hz, 1H, H-4′), 4.95 (dd, *J* = 9.8, *J* = 8.6 Hz, 1H, H-2′), 5.33 (d, *J* = 9.8 Hz, 1H, H-1′), 5.65 (t, *J* = 9.8 Hz, 1H, H-3′), 6.15 (d, *J* = 7.8 Hz, 1H, furyl-H), 6.60 (d, *J* = 7.8 Hz, 1H, furyl-H), 7.11–7.34 (m, 4H, Ar-H); ^13^C-NMR (DMSO-*d*_6_): δ (ppm) 14.1, 20.7, 20.8, 20.9, 21.0 (5 CH_3_), 27.1, 31.5, 38.2, 39.3 (cyclohexyl-C), 62.1 (C-5), 62.3 (C-6), 69.3 (C-4), 71.1 (C-3), 72.8 (spiro-C), 78.2 (C-2), 89.3 (C-1), 109.1–156.6 (Ar-C), 162.6 (pyrimidine-C2), 169.5, 169.8, 170.5, 172.5 (4 C=O). Analysis calc. for C_35_H_38_FN_3_O_10_S_2_ (743.82): C, 56.52; H, 5.15; N, 5.65. Found: C, 56.29; H, 5.05; N, 5.88.

*3′-(4-Fluorophenyl)-7′-(5-methylfuran-2-yl)-5′-(2,3,4-tri-O-acetyl-d-xylopyranosyl)-3′H-spiro[cyclohexane-1,2′-thiazolo[4,5-d]pyrimidine]* (**8**). Brownish powder, Yield: 72%; m.p. 133–134 °C; IR (KBr, υ, cm^−1^): 3045 (C-H aromatic), 2950 (C-H aliphatic), 1745 (C=O), 1615 (C=N); ^1^H-NMR (DMSO-*d*_6_): δ (ppm) 1.50–1.92 (m, 10H, 5CH_2_), 1.98, 2.05, 2.14, 2.34 (4s, 12H, 4CH_3_),4.12–4.16 (dd, *J* = 3.8, 10.2 Hz, 1H, H-5′′), 4.20–4.25 (dd, *J* = 11.3, 3.8 Hz, 1H, H-5′), 4.69 (m, 1H, H-4′), 5.05 (dd, *J* = 5.2, 8.8 Hz, 1H, H-2′), 5.33 (d, *J* = 9.6 Hz, 1H, H-1′), 5.59 (t, *J* = 8.8 Hz, 1H, H-3′), 6.17 (d, *J* = 7.8 Hz, 1H, furyl-H), 6.62 (d, *J* = 7.8 Hz, 1H, furyl-H), 7.15–7.38 (m, 4H, Ar-H); ^13^C-NMR (DMSO-*d*_6_): δ (ppm) 14.9, 20.2, 20.7, 20.9 (4 CH_3_), 27.5, 27.7, 40.6, 41.5 (cyclohexyl-C), 61.9 (C-5), 67.5 (C-4), 70.6 (C-3), 73.6 (spiro-C), 73.8 (C-2), 89.3 (C-1), 111.9–153.2 (Ar-C), 163.4 (pyrimidine-C2), 168.6, 169.9, 170.4 (3 C=O). Analysis calc. for C_32_H_34_FN_3_O_8_S_2_ (671.76): C, 57.22; H, 5.10; N, 6.26. Found: C, 57.51; H, 5.29 N, 6.11.

#### 3.1.5. 3′-(4-Fluorophenyl)-7′-(5-methylfuran-2-yl)-5′-(d-glycopyranosyl)-3′*H*-spiro[cyclohexane-1,2′-thiazolo[4,5-*d*]pyrimidine] (**9**,**10**)

The acetylated glycoside **7** or **8** (0.01 mol) was dissolved in dry methanol saturated with ammonia gas (20 mL) at 0 °C and the solution was stirred at room temperature for 8 h. The solvent was evaporated under reduced pressure at 40 °C and the residue was treated with diethyl ether (15 mL) to afford compound **9** or **10**, respectively.

*5′-(d-Glucopyranosyl)-3′-(4-fluorophenyl)-7′-(5-methylfuran-2-yl)-3′H-spiro[cyclohex-ane-1,2′-thiazolo[4,5-d]pyrimidine]* (**9**) Brownish powder, Yield: 70%; m.p. 143–145 °C; IR (KBr, υ, cm^−1^): 3488–3390 (OH), 3038 (C-H aromatic), 2948 (C-H aloiphatic), 1610 (C=N). ^1^H-NMR (DMSO-*d*_6_, δ, ppm): 1.55–1.92 (m, 10H, 5CH_2_), 2.31 (s, 3H, CH_3_),3.47–3.75 (m, 2H, H-6′, H-6′′), 3.90–4.07 (m, 1H, H-5′), 4.15–4.119 (m, 1H, H-4′), 4.72–4.89 (m, 3H, H-3′, H-2′, OH), 5.12–5.18 (m, 2H, 2OH), 5.40 (m, 1H, OH), 5.77 (d, *J* = 9.8 Hz, 1H, H-1′), 6.15 (d, *J* = 7.8 Hz, 1H, furyl-H), 6.60 (d, *J* = 7.8 Hz, 1H, furyl-H), 7.15 (d, *J* = 8.4 Hz, 2H, Ar-H), 7.38 (d, *J* = 8.4 Hz, 2H, Ar-H); Analysis calc. for C_27_H_30_FN_3_O_6_S_2_ (575.67): C, 56.33; H, 5.25; N, 7.30. Found: C, 56.02; H, 5.05; N, 7.61.

*3′-(4-Fluorophenyl)-7′-(5-methylfuran-2-yl)-5′-(d-xylo)-3′H-spiro[cyclohexane-1,2′-thiazolo[4,5-d]pyrimidine]* (**10**). Brownish powder, Yield: 69%; m.p. 143–145 °C; IR (KBr, υ, cm^−1^): 3475–3405 (OH), 3060 (C-H aromatic), 2935 (C-H aloiphatic), 1612 (C=N). ^1^H-NMR (DMSO-*d*_6_, δ, ppm): 1.54–1.93 (m, 10H, 5CH_2_), 2.32 (s, 3H, CH_3_), 3.59–3.79 (m, 2H, H-5′, H-5′′), 4.22–4.27 (m, 1H, H-4′), 4.75–4.92 (m, 3H, H-3′, H-2′, OH), 5.12–5.18 (m, 1H, OH), 5.35 (m, 1H, OH), 5.79 (d, *J* = 9.8 Hz, 1H, H-1′), 6.18 (d, *J* = 7.8 Hz, 1H, furyl-H), 6.61 (d, *J* = 7.8 Hz, 1H, furyl-H), 7.16 (d, *J* = 8.4 Hz, 2H, Ar-H), 7.39 (d, *J* = 8.4 Hz, 2H, Ar-H); Analysis calc. for C_26_H_28_FN_3_O_5_S_2_ (545.64): C, 57.23; H, 5.17; N, 7.70. Found: C, 57.44; H, 5.27; N, 7.92.

#### 3.1.6. Synthesis of Aryl 1-Thia-4-azaspiro[4.5]decan-3-ylidene)amino)-1,3,4-thiadiazole-2-thiol (**12**,**13**)

To a solution of compound **2** or **3** (0.01 mol) and 2-amino-1,3,4-thiadiazole-5-thiol (**11**) (0.01 mol) in absolute ethanol (30 mL) was added 2 mL of glacial acetic acid. The reaction mixture was refluxed for 5 h, then left to cool; the formed solid was filtered off, washed with water, and crystallized to give compounds **12** or **13**, respectively.

*5-((4-(4-Fluorophenyl)-1-thia-4-azaspiro[4.5]decan-3-ylidene)amino)-1,3,4-thiadiazole-2(3H)-thione* (**12**). Pale brown powder, Yield: 71%; m.p. 156–157 °C; IR (KBr, υ, cm^−1^): 3250 (NH), 3055 (C-H aromatic), 2930 (C-H aliphatic), 1610 (C=N). ^1^H-NMR (DMSO-*d*_6_, δ, ppm): 1.45–1.95 (m, 10H, 5CH_2_), 3.60 (s, 2H, CH_2_), 7.13–7.60 (m, 4H, Ar-H), 13.25 (s, 1H, NH); ^13^C-NMR: 23.4, 28.2, 32.0, 39.1 (cyclohexyl-C and thiadiazole CH_2_), 74.05 (spiro-C), 116.4–132.2 (Ar-C), 161.6–162.5 (thiazole-C4 and thiadiazole-C), 182.13 (C=S); MS, *m*/*z* (%): 381 (M^+^ + 1, 22); Analysis calc. for C_16_H_17_FN_4_S_3_ (380.52): C, 50.50; H, 4.50; N, 14.72. Found: C, 50.28; H, 4.37; N, 14.98.

*5-((4-(4-Fluorophenyl)-8-methyl-1-thia-4-azaspiro[4.5]decan-3-ylidene)amino)-1,3,4-thiadiazole-2(3H)-thione* (**13**). Yellow powder, Yield: 71%; m.p. 145–146 °C; IR (KBr, υ, cm^−1^): 3275 (NH), 3050 (C-H aromatic), 2938 (C-H aliphatic), 1612 (C=N). ^1^H-NMR (DMSO-*d*_6_, δ, ppm): 0.82 (s, 3H, CH_3_), 1.52–1.99 (m, 9H, cyclohexyl-H), 3.65 (s, 2H,CH_2_), 7.06–7.32 (m, 4H, Ar-H), 13.11 (s, 1H, NH); ^13^C-NMR: 15.3 (CH_3_), 22.7, 32.5, 33.6, 39.2 (cyclohexyl-C and thiadiazole CH_2_), 74.1 (spiro-C), 112.3–148.9 (Ar-C), 162.6–163.1 (thiazole-C4 and thiadiazole-C), 182.1 (C=S); MS, *m*/*z* (%): 394 (M^+^, 18); Analysis calc. for C_17_H_19_FN_4_S_3_ (394.55): C, 51.75; H, 4.85; N, 14.20. Found: C, 51.49; H, 4.77; N, 14.03.

#### 3.1.7. Synthesis of *N*-(5-(Acetylated sugar)-1,3,4-thiadiazol-2-yl)-4-(4-fluorophenyl)-1-thia-4-azaspiro[4.5]decan-3-imine (**14**–**17**)

A stirred solution of 2,3,4,6-tetra-*O*-acetyl-α-d-gluco- or 2,3,4-tri-*O*-acetyl-d-xylopyranosyl bromide (0.005 mol) in acetone (20 mL) was added portion wise to a solution of thione derivative **12** or **13** (0.005 mol) in aqueous potassium hydroxide [0.01 mol in distilled water (2 mL)]. The resulting mixture was stirred at room temperature for 5–6 h (TLC, Pet. ether/EtOAc 3:1). The solvent was evaporated under reduced pressure then ice cold water was added to the residue with stirring for 1 h. It was then left to stand overnight in a refrigerator to afford a product which was dried and crystallized from ethanol to give compound **14**–**17**.

*4-(4-Fluorophenyl)-N-(5-(2,3,4,6-tetra-O-acetyl-d-glucopyranosyl)-1,3,4-thiadiazol-2-yl)-1-thia-4-azaspiro[4.5]decan-3-imine* (**14**). Brownish solid, Yield: 68%, m.p. 138–139 °C; IR (KBr, cm^−1^): 3062 (C-H aromatic), 2936 (C-H aliphatic), 1752 (C=O), 1618 (C=N). ^1^H-NMR (DMSO-*d*_6_, δ, ppm): 1.28–1.93 (m, 10H, 5CH_2_), 1.96, 1.97, 1.99, 2.02 (4s, 12H, 4CH_3_), 3.54 (s, 2H, CH_2_), 3.67–3.71 (m, 1H, H-5′), 4.01–4.04 (dd, 1H, *J* = 3.3, *J* = 11.6 Hz, H-6′), 4.18–4.21 (dd, 1H, *J* = 11.6, *J* = 2.8 Hz, H-6′′), 4.35 (m, 1H, H-4′), 4.90–4.96 (t, 1H, *J* = 10.8 H-2′), 5.29 (d, 1H, *J* = 10.8 Hz, H-1′), 5.34 (t, 1H, *J* = 9.2 Hz, H-3′), 7.12–7.27 (m, 4H, Ar-H); ^13^C-NMR (DMSO-*d*_6_): 20.2, 20.7, 20.9, 21.1 (4 CH_3_), 23.5, 28.9, 32.0, 38.1 (cyclohexyl-C and thiadiazole CH_2_), 61.7 (C-5), 67.8 (C-6), 69.1 (C-4), 70.5 (C-3), 72.9 (spiro-C), 73.6 (C-2), 91.2 (C-1), 116.2–152.2 (Ar-C), 162.9–163.2 (thiazole-C4 and thiadiazole-C), 169.4, 169.7, 170.0, 170.5 (4 C=O). Analysis calc. for C_30_H_35_FN_4_O_9_S_3_ (710.81): C, 50.69; H, 4.96; N, 7.88. Found: C, 50.49; H, 4.82; N, 7.60.

*4-(4-Fluorophenyl)-N-(5-(2,3,4,6-tri-O-acetyl-d-xylopyranosyl)-1,3,4-thiadiazol-2-yl)-1-thia-4-azaspiro[4.5]decan-3-imine* (**15**). Gray powder Yield: 74%, m.p. 145–146 °C; IR (KBr, cm^−1^): IR (KBr, cm^−1^): 3048 (C-H aromatic), 2935 (C-H aliphatic), 1752 (C=O), 1612 (C=N); ^1^H-NMR (DMSO-*d*_6_, δ, ppm): 1.26–1.94 (m, 10H, 5CH_2_), 1.97, 1.99, 2.02 (3s, 9H, 3CH_3_), 3.52 (s, 2H, CH_2_), 4.04–4.14 (m, 2H, H-5′′, H-5′), 4.49–4.52 (dd, 1H, *J* = 6.6 Hz, *J* = 7.8 Hz, H-4′), 4.99–5.02 (dd, 1H, *J* = 7.8, *J* = 10.2 Hz Hz, H-2′), 5.29 (d, 1H, *J* = 10.2 Hz, H-1′), 5.46 (t, 1H, *J* = 7.8 Hz, H-3′), 7.27 (m, 4H, Ar-H); Analysis calc. for C_27_H_31_FN_4_O_7_S_3_ (638.74): C, 50.77; H, 4.89; N, 8.77; Found: C, 50.48; H, 4.69; N, 8.60.

*4-(4-Fluorophenyl)-N-(5-(2,3,4,6-tetra-O-acetyl-d-glucopyranosyl)-1,3,4-thiadiazol-2-yl)-8-methyl-1-thia-4-azaspiro[4.5]decan-3-imine* (**16**). Brownish solid, Yield: 65%, m.p. 136–137 °C; IR (KBr, cm^−1^): 3055 (C-H aromatic), 2940 (C-H aliphatic), 1748 (C=O), 1616 (C=N). ^1^H-NMR (DMSO-*d*_6_, δ, ppm): 0.83 (d, 3H, *J* = 5.8 Hz, CH_3_), 1.28–1.89 (m, 9H, cyclohexyl-H), 1.95, 1.97, 2.00, 2.06 (4s, 12H, 4CH_3_), 3.50 (s, 2H, CH_2_), 3.71–3.75 (m, 1H, H-5′), 4.15–4.19 (m, 1H, H-6′′), 4.25–4.31 (dd, 1H, *J* = 3.4, *J* = 10.8 Hz, H-6′), 4.40 (dd, 1H, *J* = 8.2 Hz, *J* = 9.8 Hz, H-4′), 5.08 (t, 1H, *J* = 10.8 H-2′), 5.31 (d, 1H, *J* = 10.4 Hz, H-1′), 5.66 (t, 1H, *J* = 9.2 Hz, H-3′), 7.11–7.23 (m, 4H, Ar-H); ^13^C-NMR (DMSO-*d*_6_): 17.7, 20.8, 20.8, 21.1, 21.1 (5 CH_3_), 27.3, 31.1, 38.5, 38.7 (cyclohexyl-C and CH_2_), 62.1 (C-5), 67.7 (C-6), 68.8 (C-4), 71.1 (C-3), 72.8 (spiro-C), 73.8 (C-2), 89.2 (C-1), 116.3–152.2 (Ar-C), 162.1–163.4 (thiazole-C4 and thiadiazole-C), 169.5, 169.7, 170.5, 171.8 (4 C=O). Analysis calc. for C_31_H_37_FN_4_O_9_S_3_ (724.83): C, 51.37; H, 5.15; N, 7.73. Found: C, 51.05; H, 4.96; N, 7.98.

*4-(4-Fluorophenyl)-N-(5-(2,3,4,6-tri-O-acetyl-d-xylopyranosyl)-1,3,4-thiadiazol-2-yl)-8-methyl-1-thia-4-azaspiro[4.5]decan-3-imine* (**17**). Pale yellow powder, Yield: 75%, m.p. 136–137 °C; IR (KBr, cm^−1^): 3040 (C-H aromatic), 2938 (C-H aliphatic), 1750 (C=O), 1617 (C=N); ^1^H-NMR (DMSO-*d*_6_, δ, ppm): 0.89 (d, 3H, *J* = 5.6 Hz, CH_3_), 1.29–1.95 (m, 9H, cyclohexyl-H), 1.99, 2.03, 2.05 (3s, 9H, 3CH_3_), 3.53 (s, 2H, CH_2_), 4.02–4.14 (m, 2H, H-5′′, H-5′), 4.76–4.79 (dd, 1H, *J* = 7.0 Hz, *J* = 7.6 Hz, H-4′), 5.15 (dd, 1H, *J* = 7.5, *J* = 9.8 Hz, H-2′), 5.30 (d, 1H, *J* = 9.8 Hz, H-1′), 5.39 (t, 1H, *J* = 7.5 Hz, H-3′), 7.14–7.27 (m, 4H, Ar-H); ^13^C-NMR (DMSO-*d*_6_): 17.0, 20.9, 21.1, 21.9 (4 CH_3_), 24.5, 28.8, 32.8, 38.4 (cyclohexyl-C and CH_2_), 67.8 (C-5), 68.1 (C-4), 70.4 (C-3), 73.6 (spiro-C), 74.1 (C-2), 89.5 (C-1), 116.4–154.9 (Ar-C), 160.9–163.4 (thiazole-C4 and thiadiazole-C), 169.7, 170.4, 171.8 (3 C=O); Analysis calc. for C_28_H_33_FN_4_O_7_S_3_ (652.77): C, 51.52; H, 5.10; N, 8.58. Found: C, 51.29; H, 5.02; N, 8.39.

#### 3.1.8. 4-(4-Fluorophenyl)-*N*-(5-(d-glycopyranosyl)-1,3,4-thiadiazol-2-yl)-8-methyl-1-thia-4-azaspiro[4.5]decan-3-imine (**18**,**19**)

The acetylated 1,3,4-thiadiazolyl glycoside **16** or **17** ((0.005 mol)) was dissolved with stirring in anhydrous methanol saturated with ammonia gas (20 mL) at 0 °C and the solution was then further stirred at room temperature for 7 h. The solvent was evaporated under reduced pressure at 40 °C, then a mixture of diethyl ether and pet. ether (1:1) was added to the residue with stirring for about 30 min. The formed solid substance was filtered, washed with cold ethanol and crystallized to give **18** or **19**, respectively.

*4-(4-Fluorophenyl)-N-(5-(d-glucopyranosyl)-1,3,4-thiadiazol-2-yl)-8-methyl-1-thia-4-azaspiro[4.5]decan-3-imine* (**18**). Pale yellow powder, Yield: 69%, m.p. 180–181 °C; IR (KBr, cm^−1^): 3483–3428 (OH), 3058 (C-H aromatic), 2925 (C-H aliphatic), 1615 (C=N); ^1^H-NMR (DMSO-*d*_6_, δ, ppm): 0.88 (d, 3H, *J* = 5.8 Hz, CH_3_),1.47–2.07 (m, 9H, cyclohexyl-H), 3.43–3.51 (m, 4H, H-6′′, H-6′ and CH_2_), 3.64–3.69 (m, 1H, H-5′), 3.75–4.18 (m, 3H, H-4′, H-3′ and OH), 4.21–4.23 (m, 1H, OH), 5.11 (m, 2H, OH and H-2′), 5.33 (m, 1H, OH), 5.65 (d, 1H, *J* = 9.8 Hz, H-1′), 7.16–7.61 (m, 4H, Ar-H); ^13^C-NMR (DMSO-*d*_6_): 16.4 (CH_3_), 25.9, 28.8, 39.1 (cyclohexyl-C and CH_2_), 62.1 (C-6), 67.8 (C-5), 68.9 (C-4), 71.1 (C-3), 72.5 (spiro-C), 78.2 (C-2), 90.2 (C-1), 109.2–154.2 (Ar-C), 160.5–163.1 (thiazole-C4 and thiadiazole-C); Analysis calc. for C_23_H_29_FN_4_O_5_S_3_ (556.69): C, 49.62; H, 5.25; N, 10.06. Found: C, 49.89; H, 4.99; N, 9.95.

*4-(4-Fluorophenyl)-N-(5-(d-xylopyranosyl)-1,3,4-thiadiazol-2-yl)-8-methyl-1-thia-4-azaspiro[4.5]decan-3-imine* (**19**). Pale yellow powder, Yield: 63%, m.p.170–171 °C; IR (KBr, cm^−1^): 3495–3446 (OH), 3060 (C-H aromatic), 2918 (C-H aliphatic), 1619 (C=N); ^1^H-NMR (DMSO-*d*_6_, δ, ppm): 0.89 (d, 3H, *J* = 5.8 Hz, CH_3_), 1.47–2.14 (m, 9H, cyclohexyl-H), 3.39–3.44 (m, 2H, H-5′′, H-5′), 3.52 (s, 2H, CH_2_), 3.65–3.88 (m, 2H, H-4′, H-3′), 4.23–4.27 (m, 2H, 2OH), 4.90–5.12 (m, 2H, H-2′, OH), 5.65 (d, 1H, *J* = 9.8 Hz, H-1′), 6.87–7.58 (m, 4H, Ar-H); Analysis calc. for C_22_H_27_FN_4_O_4_S_3_ (526.66): C, 50.17; H, 5.17; N, 10.64. Found: C, 49.98; H, 5.02; N, 10.50.

### 3.2. Anticancer Screening

#### 3.2.1. Cells

Cell culture of HepG-2 (human liver carcinoma), PC-3 (human prostate adenocarcinoma) and HCT116 (human colorectal carcinoma) cell lines were purchased from the American Type Culture Collection (Rockville, MD, USA) and maintained in Roswell Park Memorial Institute (RPMI-1640) medium which was supplemented with 10% heat-inactivated FBS (fetal bovine serum), 100 U/mL penicillin and 100 U/mL streptomycin. The cells were grown at 37 °C in a humidified atmosphere of 5% CO_2_.

#### 3.2.2. MTT Cytotoxicity Assay

The antitumor activity against liver HepG-2, prostate PC-3 and colon HCT-116 human cancer cell lines was estimated using the 3-[4,5-dimethyl-2-thiazolyl)-2,5-diphenyl-2*H*-tetrazolium bromide (MTT) assay, which is based on the cleavage of the tetrazolium salt by mitochondrial dehydrogenases in viable cells [44,45,46]. Cells were dispensed in a 96-well sterile microplate (5 × 10^4^ cells/well), and incubated at 37 °C with series of different concentrations, in DMSO, of each tested compound or Doxorubicin^®^ (positive control) for 48 h in a serum-free medium prior to the MTT assay. After incubation, media were carefully removed, 40 µL of MTT (2.5 mg/mL) was added to each well and then incubated for an additional 4 h. The purple formazan dye crystals were solubilized by the addition of 200 µL of DMSO. The absorbance was measured at 590 nm using a SpectraMax^®^ Paradigm^®^ Multi-Mode microplate reader. The relative cell viability was expressed as the mean percentage of viable cells compared to the untreated control cells.

#### 3.2.3. Statistical Analysis

All experiments were conducted in triplicate and repeated on three different days. All the values were represented as mean ± SD. IC_50_s were determined by probit analysis using the SPSS software program (version 20, SPSS Inc., Chicago, IL, USA).

## 4. Conclusions

The thioglycoside derivatives of novel 1,3,4-thiadiazole and thiazolopyrimidine compounds incorporating 1-thia-azaspiro[4.5]decane ring system were synthesized. The anticancer activity against human liver hepatocellular carcinoma (HepG-2), human prostate adenocarcinoma (PC-3) and human colorectal carcinoma (HCT-116) cell lines was investigated. A number of compounds showed moderate to high anticancer activity and the results revealed the importance of attachment of glycosyl moieties to the thiazolopyrimidine or (1,3,4-thiadiazolyl)thiazolidinone ring systems.

## Figures and Tables

**Table 1:** The anticancer IC_50_ values of compounds using a MTT assay against HepG-2, PC-3 and HCT116 cell lines.

Compound	HCT-116	PC-3	HepG-2
	IC_50_ (nM) ± SD		
**1**	178.1 ± 5.3	184.1 ± 6.3	509.5 ± 9.2
**3**	201.3 ± 3.8	210.2 ± 4.7	541.4 ± 7.2
**7**	120.1 ± 4.1	223.9 ± 6.4	134.5 ± 4.9
**9**	95.8 ± 3.7	281.8 ±3.6	285.1 ± 3.9
**12**	250.3 ± 5.7	437.4 ± 5.3	425.6 ± 8.6
**13**	204.6 ± 3.8	217.7 ± 8.1	581.5 ± 9.3
**14**	92.2 ± 1.8	120.8 ± 3.9	176.1 ± 2.9
**15**	204.6 ± 3.5	331.3 ± 9.2	348.5 ± 7.4
**17**	127.4 ± 5.6	693.9 ± 8.9	209.7 ± 10.1
**18**	106.3 ± 2.9	111.6 ± 3.8	334.1 ± 9.9
**19**	106.6 ± 4.2	9493.8 ± 20.9	599.0 ± 11.5
Doxorubicin	126.7 ± 8.9	129.7 ± 2.4	117.1 ± 5.8
